# The RNA Capping Enzyme Domain in Protein A is Essential for Flock House Virus Replication

**DOI:** 10.3390/v10090483

**Published:** 2018-09-09

**Authors:** Tania Quirin, Yu Chen, Maija K. Pietilä, Deyin Guo, Tero Ahola

**Affiliations:** 1 Department of Microbiology, Faculty of Agriculture and Forestry, University of Helsinki, Viikinkaari 9 (P.O. Box 56), 00014 Helsinki, Finland; tania.quirin@helsinki.fi (T.Q.); maija.pietilae@uzh.ch (M.K.P.); 2 State Key Laboratory of Virology, Modern Virology Research Center, College of Life Sciences, Wuhan University, Wuhan 430072, China; chenyu@whu.edu.cn; 3 School of Medicine, Sun Yat-sen University, Guangzhou 510080, China; guodeyin@mail.sysu.edu.cn

**Keywords:** RNA replication, nodavirus, alphavirus, RNA capping enzyme, replication complex

## Abstract

The nodavirus flock house virus (FHV) and the alphavirus Semliki Forest virus (SFV) show evolutionarily intriguing similarities in their replication complexes and RNA capping enzymes. In this study, we first established an efficient FHV trans-replication system in mammalian cells, which disjoins protein expression from viral RNA synthesis. Following transfection, FHV replicase protein A was associated with mitochondria, whose outer surface displayed pouch-like invaginations with a ‘neck’ structure opening towards the cytoplasm. In mitochondrial pellets from transfected cells, high-level synthesis of both genomic and subgenomic RNA was detected in vitro and the newly synthesized RNA was of positive polarity. Secondly, we initiated the study of the putative RNA capping enzyme domain in protein A by mutating the conserved amino acids H93, R100, D141, and W215. RNA replication was abolished for all mutants inside cells and in vitro except for W215A, which showed reduced replication. Transfection of capped RNA template did not rescue the replication activity of the mutants. Comparing the efficiency of SFV and FHV trans-replication systems, the FHV system appeared to produce more RNA. Using fluorescent marker proteins, we demonstrated that both systems could replicate in the same cell. This work may facilitate the comparative analysis of FHV and SFV replication.

## 1. Introduction

Flock house virus (FHV), a member of the family *Nodaviridae*, was first isolated from a grass grub (*Costelytra zealandica*) in New Zealand [1]. It is a small non-enveloped virus comprising an icosahedral protein capsid and a single-stranded RNA genome of positive polarity [2,3]. Studies on FHV infection and the replication of its genome have provided valuable insights about virus entry and assembly [3], and antiviral immune response [4]. FHV has recently been used as a platform for the development of novel vaccines [5,6,7,8,9].

**Figure 1:**
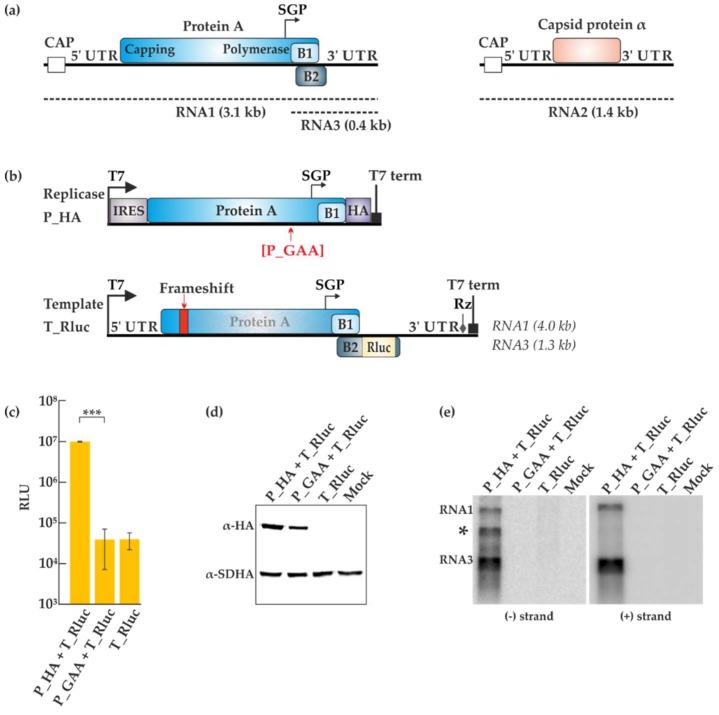
Trans-replication of flock house virus (FHV). (**a**) Depiction of the bipartite FHV genome. Both RNA1 and RNA2 are capped, positive sense RNAs flanked by untranslated regions (UTRs). The replicase protein A is translated from RNA1. The subgenomic RNA3 arises from the subgenomic promoter (SGP) and is used for the production of the small proteins B1 and B2. RNA2 codes for the capsid protein α. The dotted lines denote the complementary negative strands generated during replication. (**b**) The plasmids for the trans-replication system produce RNAs from the T7 polymerase promoter, ending with the T7 terminator (T7 term) and/or ribozyme (Rz) elements. The locations for the polymerase inactivating mutation and the frameshift mutation are shown in red. (**c**) BSR T7/5 cells were transfected with the indicated plasmids and luciferase activity was measured 40 h post-transfection. Luciferase signals are shown as mean (corrected for mock transfected background) from two independent experiments. Error bars represent the standard deviation. *** designates *p* < 0.001 (Student’s *t* test). RLU: relative light units. (**d**) Replicase expression following transfections in BSR T7/5 cells was detected by Western blotting with anti-hemagglutinin (HA) antibodies. The expression of the cellular protein succinate dehydrogenase (SDHA) is shown as a control. (**e**) Viral RNA synthesis was analyzed by Northern blotting using probes designed to detect negative and positive strands RNAs. The asterisk marks an uncharacterized RNA product that was sometimes observed during minus strand detection. Rluc: Renilla luciferase

The FHV genome is bipartite (Figure 1a). Both RNA1 (3.1 kb) and RNA2 (1.4 kb) are flanked by untranslated regions (UTRs), are capped but not polyadenylated, and act as mRNA [9]. Protein A (998 amino acids (aa)) is translated from RNA1 and is a prerequisite for viral replication to occur. The C-terminal part of protein A exhibits motifs typical for RNA-dependent RNA polymerases (RdRps) and the polymerase activity of the protein has been demonstrated [10]. During virus replication, a subgenomic RNA3 is produced, from which proteins B1 and B2 are expressed. While the functions of B1 are unclear, protein B2 contains a double-stranded RNA (dsRNA) binding domain and has been shown to act as a silencing suppressor during insect cell infection [4]. RNA2 encodes the capsid protein α. Although FHV only infects insects in nature, the viral RNA replication process can take place in many cell types including mammalian cells, plant cells, and even the baker’s yeast *Saccharomyces cerevisiae* [11,12,13]. Thus, the host factors necessary for FHV replication must be generally available, which makes it an attractive, versatile model system [14]. 

Insect nodaviruses have been classified in the genus *Alphanodavirus*, whereas fish nodaviruses, some of which are economically important pathogens in aquaculture, have been classified in the genus *Betanodavirus*. However, recent studies indicate that nematodes and other organisms have nodavirus-like viruses [15]. Based on the similarities of RdRp sequences, metagenomic surveys of RNA viruses have placed nodaviruses and their relatives together with another group of simple positive-strand RNA viruses, the plant tombusviruses and their relatives [16,17]. This still rather tentative large group or superfamily of RNA viruses is called the tombus–noda clade. All positive-strand viruses replicate their RNA in association with cellular membranes [18], and interestingly both the tombusviruses and the nodaviruses induce similar, small membrane invaginations (termed spherules) as their replication sites [19,20,21].

The alphavirus-like superfamily is a separate, well-characterized superfamily of positive-strand RNA viruses, whose existence and monophyly are supported by the recent metagenomic studies [17,22]. Although there is no close relationship between the RdRps of alpha-like viruses and nodaviruses, these groups unexpectedly share a conserved domain at the N-terminus of their replicase proteins [23]. In the animal alphaviruses, it has been biochemically characterized as the enzyme responsible for viral RNA capping, and, therefore, we will refer to it as the RNA capping enzyme domain [23,24,25], although we stress that this activity has not been established for the FHV protein. The capping enzyme does not exist in the tombusviruses, whose genomes are uncapped. Thus, it is possible that the shared capping enzyme domain represents ancient lateral gene transfer between alpha-like virus and nodavirus ancestors. 

We have studied the replication complexes and replication enzymes of alphaviruses [26]. Intriguingly, the alphavirus replication sites are also characterized as spherule invaginations on membranes, which look quite similar to the nodavirus spherules in electron microscopic images and low-resolution tomographic reconstructions [19,27,28,29]. To understand the deep roots of RNA virus evolution, it would, therefore, be crucial to study, whether the spherules of alpha-like viruses and noda–tombus-like viruses have only superficial similarity that arose via convergent evolution to fulfill similar needs. Alternatively, it remains possible that they could display underlying structural and functional similarities. In recent years, we have extensively used trans-replication systems to characterize the replication complex spherules of alphaviruses [30]. In these systems, the replicase proteins are expressed from a plasmid construct, and a replication-competent template RNA is produced from a second plasmid. Therefore, the two components can be independently modified to study, for instance, the requirements for spherule formation [31,32]. 

To facilitate the comparison between alphaviruses and FHV, we report here an efficient FHV trans-replication system in mammalian cells, which is exactly analogous to the alphavirus system. As a result, the replication of FHV and Semliki Forest virus (SFV) can now be compared side by side to understand whether the spherules are formed by different or similar processes e.g., in terms of host factor requirements. Secondly, we used the trans-replication system for initial characterization of the conserved, putative RNA capping enzyme domain in protein A through mutational analysis. Alteration of the most conserved residues in the putative capping domain completely abolished RNA synthesis. 

## 2. Materials and Methods 

### 2.1. Plasmid Constructs

Plasmid containing the sequence of FHV RNA1 was a kind gift from Dr. Xi Zhou (Wuhan University, China). All the constructs used in this study are under the bacteriophage T7 promoter (Figure 1b). The P_HA replicase construct consists of an internal ribosome entry site (IRES) element followed by FHV protein A, a spacer (GGSGGSGG), the influenza hemagglutinin (HA) tag and the T7 terminator. P_GAA replicase construct was created with the same features as P_HA except that a double mutation was introduced at positions 692/693 ((DD to AA) of protein A, resulting in an inactivated polymerase. The replicase construct P_GAA_Vis was made by adding a second open reading frame after the T7 terminator of P_GAA, encoding the fluorescent marker mCherry fused to a nuclear localization signal (NLS), driven by a second the T7 promoter. P_GAA_Vis was used as a control for correlative light electron microscopy (CLEM) experiments to visualize the expression of the polymerase defective protein after transfection, analogous to Kallio et al. [33]. The T_Rluc template construct is flanked by FHV UTRs and contains the protein A sequence and Renilla luciferase (after aa 100 of protein B2) under the control of the subgenomic promoter. A frameshift was introduced in protein A as described by Miller et al. [12] to prevent protein A expression. Briefly, the complementary DNA (cDNA) of RNA1 was digested at position 373 of RNA1, filled in with a T4 DNA polymerase and re-ligated. The template T_eGFP was constructed by substituting Renilla luciferase with enhanced green fluorescent protein (eGFP). The template FHV_T was created by cutting out GFP from T_eGFP using the restriction enzyme *Nar*I. The replicase capping mutants H93A, R100A, D141A and W215A were generated using site-directed mutagenesis by polymerase chain reaction (PCR). The Δ2-35A mutant was generated by introducing a *NcoI* site at position 35 of protein A during PCR followed by restriction digestion. The SFV replicase constructs used have been previously described [30,34]. 

### 2.2. Cells, Transfection and Renilla Luciferase Assay

BSR T7/5 cells [35] were grown at 37 °C in a cell culture medium consisting of Dulbecco’s modified Eagle’s medium supplemented with 10% fetal bovine serum (Gibco, Baltimore, MD, USA), 2% bacto tryptose phosphate broth (Difco, Becton, Dickinson and Company, Franklin Lakes, NJ, USA), 2 mM l-glutamine, 1% non-essential amino acids, 1 mg/mL G418, 100 U/mL penicillin and 100 µg/mL streptomycin (Gibco) [35]. Polyethylenimine (PEI), linear molecular weight (MW) 25,000 (Polysciences, Inc., Warrington, PA, USA) was used for the transfection of FHV constructs into BSR T7/5 cells at a 2:1 ratio of DNA to PEI in 150 mM NaCl. PEI was dissolved in sterile water to a final concentration of 1 mg/mL, the pH was adjusted to 7.2 with 1 M hydrochloric acid, sterilized by filtration through a 0.22 µm cellulose acetate filter and stored at −80 °C. Three cycles of freezing and thawing were done before using the PEI transfection mixture. All transfections involving the SFV constructs were done using Lipofectamine LTX & PLUS (Thermo Fisher Scientific, Waltham, MA, USA) and incubated at 37 °C. For 96-well plates, each well was co-transfected with 50 ng of FHV replicase plasmid and 50 ng of FHV template plasmid or 40 ng of SFV replicase plasmid and 50 ng of SFV template plasmid. The total amount of plasmid transfected was scaled up according to the surface area of the plate used. Following transfections with FHV, cells were incubated at 28 °C up to 40 h. The medium was replaced by cell culture medium at 20 h. The transfection of SFV constructs has been described previously [32]. At 40 h (FHV) or 16 h (SFV) post-transfection, the cells were lysed with Renilla luciferase (Rluc) assay lysis buffer (Promega, Madison, WI, USA) and luminescence was measured using Rluc Assay Kit (Promega) as per the manufacturer’s instructions.

### 2.3. Antibodies

Mouse monoclonal antibody against the flavoprotein subunit of the succinate dehydrogenase involved in mitochondrial complex II (anti-SDHA) was purchased from Abcam. Mouse monoclonal against the human influenza virus hemagglutinin tag (anti-HA tag) was purchased from Sigma-Aldrich. Rabbit polyclonal anti-Tom20 (Santa Cruz Biotechnology Inc., Dallas, TX, USA) was used as a primary antibody for the detection of mitochondria in the immunofluorescence experiments. Mouse monoclonal J2 antibody (Scion, Budapest, Hungary) was used for the detection of dsRNA. Secondary antibodies used for immunofluorescence and conjugated with Alexa Fluor 488/568/647 were obtained from Molecular Probes (Invitrogen, Carlsbad, CA, USA). IRDye 800 CW donkey anti-mouse IgG (H + L) secondary antibodies (LI-COR Biosciences, Lincoln, NE, USA) were used for Western blotting.

### 2.4. Western Blotting

BSR T7/5 cells, grown at 37 °C to confluency in 6-well plates, were co-transfected with 1.25 µg of plasmid expressing FHV protein A and 1.25 µg of plasmid producing the FHV RNA template. 40 h post-transfection, the cells were washed with cold phosphate-buffered saline (PBS) and lysed in 2 × Laemmli buffer containing β-mercaptoethanol. The proteins were separated on a 10% sodium dodecyl sulphate–polyacrylamide gel electrophoresis (SDS-PAGE) gel and transferred to a Hybond ECL nitrocellulose membrane (GE Healthcare Life Sciences, Chicago, IL, USA). The nitrocellulose membrane was blocked in Tris-buffered saline (TBS) and 5% skim milk for 2 h at room temperature. The membrane was then incubated with anti-HA tag and anti-SDHA antibodies in Tris-buffered saline containing 0.1% Tween and 5% skim milk for an hour and washed several times to remove the unbound the antibodies. Detection was done by incubating the membrane with secondary antibodies for an hour at room temperature, followed by several washes. The proteins were visualized using Odyssey infrared imaging system (LI-COR Biosciences).

### 2.5. Isolation of Crude Mitochondrial Pellet

Following transfection, BSR T7/5 cells were washed once with PBS, detached using a cell scraper and transferred to a 50 mL Falcon tube. The cell suspension was centrifuged at 600× *g* for 10 min at 4 °C. The supernatant was discarded and the cells were re-suspended in 3 mL homogenisation buffer (10 mM Tris-MOPS (3-(*N*-morpholino)propanesulfonic acid), pH 7.4; 1 mM ethylene glycol tetraacetic acid (EGTA); 200 mM sucrose; Pierce protease inhibitor, ethylenediaminetetraacetic acid (EDTA)-free (Thermo Fisher Scientific); 0.2 U/µL RiboLock). Homogenisation was done with a 7 mL Kimble Chase glass dounce tissue grinder (Sigma-Aldrich, St. Louis, MS, USA) with a glass B pestle until approximately 90% of the cells appeared lysed under the light microscope. The lysate was centrifuged at 600× *g* for 10 min at 4 °C to separate the cell debris and nuclear fraction from the cytosolic fraction. The supernatant was collected and centrifuged at 7000× *g* for 10 min at 4 °C to obtain a crude mitochondrial pellet (CMP). The CMP was washed with 200 µL WS buffer (50 mM Tris-HCl, pH 7.5; Pierce protease inhibitor, EDTA-free; 0.2 U/µL RiboLock-Thermo Fisher Scientific) and resuspended in 250 µL WS buffer. To preserve the integrity of the membranes in the CMP, 15% glycerol (*v*/*v*) was added to samples before storage in aliquots at −80 °C.

### 2.6. In Vitro Transcribed RNA Template

Ten µg of T_Rluc plasmid was linearized with *Eco*RI and electrophoresed in a 1% agarose gel. The DNA band was excised from the gel and extracted using GeneJET gel extraction kit (Thermo Fisher Scientific) as per the manufacturer’s instructions. In vitro transcribed uncapped T_Rluc and FHV_T RNA were made by assembling on ice 750 ng linearized DNA, a final concentration of 1 mM (each) ATP/CTP/GTP/UTP, 5 mM dithiothreitol (DTT), transcription buffer (Promega), 0.2 U/µL RNasin ribonuclease inhibitor (Promega) and 40 U T7 RNA polymerase (Promega) in a final volume of 50 µL. In vitro transcribed capped T_Rluc and FHV_T RNA were made similarly except for an rNTP mixture comprising 1 mM ATP/CTP/UTP, 0.5 mM GTP and the addition of cap analog m^7^G(5′)ppp(5′)G (New England BioLabs, Ipswich, MA, USA) to a final concentration of 1 mM. The reaction was incubated for 2 h at 37 °C, after which 2 µL of RNase-free DNaseI (Promega) was added and further incubated for 35 min. The reaction was stopped by adding 5 µL of DNase STOP solution (Promega) and heating for 10 min at 65 °C. The newly transcribed RNA was precipitated by adding 7.5 M lithium chloride (with 50 mM EDTA, pH 8.0) to a final concentration of 2.5 M lithium chloride. The mixture was incubated overnight at −20 °C, followed by centrifugation for 20 min at 15,000× *g* at 4 °C. The supernatant was carefully removed and discarded. The RNA pellet was washed with 70% ethanol and centrifuged again. The ethanol was removed and the pellet allowed to air dry. The RNA template was resuspended in 20 µL of 1 mM sodium citrate (pH 6.4) and kept on ice. The concentration was measured with Nanodrop spectrophotometer and the integrity of the RNA was assessed by agarose gel electrophoresis.

### 2.7. Flock House Virus In Vitro Replication Assay and Agarose Gel Electrophoresis

The FHV in vitro replication assay (IVRA) described here was adapted from Short et al. [36]. Briefly, 8 µL of CMP was added to a replication buffer (50 mM Tris, pH 8.0; 18 mM MgCl_2_; 30 mM KCl; 16 mM NaCl; 250 mM sucrose; 10 µg/mL actinomycin D; 0.2 U/µL RiboLock; 1 mM ATP/GTP/UTP; 10 µM CTP; 5 µCi of CTP[α-32P] (specific activity, 3000 Ci/mmol, Perkin Elmer) to a final volume of 50 µL. The reaction was incubated for 90 min at 30 °C and the unincorporated nucleotides were removed using Micro Bio-Spin P-30 gel columns, Tris buffer, RNase-free (Bio-Rad, Berkeley, CA, USA). RNA isolation was performed as described by Scholte et al. [37]. In brief, acid phenol (Ambion) was used for the extraction following which RNA was precipitated with isopropanol, washed with 75% ethanol, and re-suspended in 20 µL of 1 mM sodium citrate (pH 6.4). A denaturing mixture (67% formamide, 23% formaldehyde, 6.7% glycerol, 13 mM MOPS (pH 7.2), 6.7 mM NaAc, 2.7 mM EDTA, 0.07% SDS, and 0.03% bromophenol blue) [37] was added to the RNA samples, incubated at 75 °C for 15 min, briefly vortexed and put on ice. RNAs were separated in a 1% denaturing formaldehyde agarose gel for 2 h at 100 V. The gel was dried and exposed to a phosphorimaging plate. Detection was performed using Typhoon Trio (GE Healthcare Life Sciences).

### 2.8. Northern Blotting

Northern blotting was performed as described in Kallio et al. [31]. In brief, BSR T7/5 cells, grown 37 °C to confluency in 6-well plates, were co-transfected with 1.25 µg of plasmid expressing FHV protein A and 1.25 µg of plasmid producing the FHV RNA template. Cells were lysed and collected with TRIsure reagent (Bioline Reagents Ltd, London, UK), followed by phenol-chloroform RNA isolation. 2 μg of total RNA isolated were separated on a 1% denaturing agarose gel and transferred to a positively charged Amersham Hybond-N+ nylon filter (GE Healthcare Life Sciences) by capillary blotting overnight. RNA was cross-linked to the membrane with Stratalinker (Stratagene, La Jolla, CA, USA). ^32^P-labeled antisense probes were made by in vitro transcription with T7 RNA polymerase from PCR-amplified DNA fragments containing sequences of the *Rluc* gene and the T7 promoter. The probe for positive-strand RNA detection corresponded to nucleotides 32 to 676, and the probe for negative-strand RNA detection corresponded to nucleotides 38 to 623 of the *Rluc* gene as present on template constructs. Hybridization was performed at 60 °C overnight with 10^6^ cpm of the RNA probe. The membrane was washed as described previously [38] and exposed to a phosphorimaging plate for 24 h. Detection was performed using Typhoon Trio (GE Healthcare Life Sciences).

### 2.9. Confocal Microscopy and Correlative Light Electron Microscopy 

BSR T7/5 cells were seeded on glass coverslips and incubated overnight at 37 °C. The cells were co-transfected with 250 ng of P_HA and 250 ng of T_eGFP using PEI reagent and the medium was replaced after 20 h. At 40 h post-transfection, the cells were fixed with 4% paraformaldehyde for 20 min, washed three times with PBS, quenched with NH_4_Cl, washed three times with Dulbecco’s PBS containing 0.2% bovine serum albumin (BSA) and permeabilized with 0.1% Triton X-100. The samples were incubated with primary antibodies for 1 h, washed three times with Dulbecco’s PBS containing 0.2% BSA and incubated with secondary antibodies for 1 h followed by further washes. ProLongGold containing DAPI (Molecular Probes, Eugene, OR, USA) was used for mounting. Imaging was done with Leica (Wetzlar, Germany) TCS SP5 confocal microscope (HCX APO 63×/1.30 Corr glycerol objective) or Leica TCS Sp5II HCS A confocal microscope (HCX PL APO 63×/1.2 WCorr/0.17 CS water objective). The images were analyzed using ImageJ software.

For correlative light electron microscopy (CLEM), 7.5 × 10^4^ BSR T7/5 cells were seeded on 35 mm glass-bottom P35G-2-14-C Grid dishes (MatTek Corporation, Ashland, MA, USA) and incubated overnight at 37 °C. Cells were co-transfected with 1 µg of P_HA + 1 µg of T_eGFP or P_GAA_Vis + T_eGFP, using PEI reagent. Transfection of P123^HA^4 + Tmed + P_HA + T_eGFP (1 µg each) was done with Lipofectamine LTX & PLUS (Thermo Fisher Scientific). For all samples, the medium was replaced by cell culture medium after 20 h. At 40 h post-transfection, the cells were fixed with 2% glutaraldehyde in 0.1 M sodium-cacodylate buffer (NaCac, pH 7.4) for 20 min at room temperature, washed three times with 0.1 M NaCac buffer and left in the last wash. Cells expressing GFP were imaged using fluorescent and differential interference contrast (DIC) settings on a Leica TCS Sp5II HCS A confocal microscope with the HC PL APO 20×/0.7 CS (air) objective lens and the gridded dishes were marked accordingly. The samples were prepared for transmission electron microscopy as previously described [39]. The positive cells observed during fluorescent and DIC imaging were located using a Jeol JEM-1400 transmission electron microscope (Jeol USA Inc, Peabody, MA, USA), and Gatan Orius (Gatan Inc., Pleasanton, CA, USA) SC 1000B bottom mounted CCD-camera was used for imaging.

### 2.10. Polarity of RNA Products Detected with Capture Probes

*Eco*RI-linearized FHV_T_Rluc and FHV_T_minus and *Xho*I-linearized pEAV221Δ [34] plasmids were used as templates for in vitro transcription. Transcripts of FHV_T_Rluc and FHV_T_minus corresponding to capture probes for minus and plus strands, respectively, and a transcript containing nucleotides 1-2042 of equine arteritis virus (EAV) genome were prepared using mMESSAGE mMACHINE T7 Transcription Kit (Ambion) according to the manufacturer’s instructions. In vitro transcription reactions to prepare ^32^P-labeled transcripts of FHV_T_Rluc and FHV_T_minus consisted of 750 ng of the respective linearized plasmid, transcription buffer (Promega), 800 U/mL T7 RNA polymerase (Promega), 1000 U/mL RiboLock (Thermo Fisher Scientific), 5 mM DTT, 1 mM ATP, UTP, GTP and CTP, and 0.133 μM α-^32^P-CTP (20 μCi) (Perkin Elmer, Ayer Rajah, Singapore). The reactions were incubated for 2 h at 37 °C followed by RQ1 RNase-free DNase treatment (40 U/mL; Promega) for 35 min at 37 °C and inactivation by RQ1 DNase STOP solution (Promega) for 10 min at 65 °C. Unincorporated label was removed using RNase-free Micro Bio-Spin P-30 Gel Columns (Bio-Rad). Hybridization with the ^32^P-labeled RNA transcripts and IVRA products was performed as described [34].

## 3. Results

### 3.1. Flock House Virus Trans-Replication System that Disassociates Viral Protein Expression and RNA Synthesis

The FHV trans-replication system was designed as depicted in Figure 1b, using bacteriophage T7 promoter, with the aim of using it in BSR T7/5 cells, a subclone of baby hamster kidney (BHK) cells expressing T7 polymerase [35]. We focused only on the replication and production of RNA1 and RNA3; RNA2 was not used in any experiment. The expression of protein A was boosted by encephalomyocarditis virus internal ribosome entry site (IRES) and the protein was fused C-terminally to influenza hemagglutinin (HA) tag, to produce the replicase plasmid termed P_HA. A second replicase plasmid producing P_GAA contained a double missense mutation (DD to AA at positions 692/693 of protein A) with the intention of inactivating the polymerase. This mutation targets one of the most conserved sites in polymerases [40]. The mRNAs for P_HA and P_GAA can be transcribed intracellularly by the T7 polymerase present in BSR T7/5 cells, but they lack the viral UTRs required for RNA replication. The template plasmid T_Rluc contains the UTRs and includes a frameshift that prevents the expression of protein A. The gene encoding Rluc was inserted under the control of the subgenomic promoter, in fusion with the B2 open reading frame. 

The replicase and template constructs were transfected into BSR T7/5 cells and luciferase activity was measured 40 h post-transfection (Figure 1c). High levels of luciferase were obtained from the P_HA + T_Rluc combination, as opposed to the background obtained from the inactive replicase P_GAA + T_Rluc combination or T_Rluc alone. Protein expression was analyzed by Western blotting as seen in Figure 1d. The inactivating polymerase mutation in P_GAA did not hinder protein A expression, whereas the frameshift mutation in the T_Rluc template prevented it as expected. The analysis of viral RNAs by Northern blotting revealed both genomic RNA1 and subgenomic RNA3 synthesis with the active replicase (Figure 1e). The amplification of the RNA by protein A is considerable, as the initial amount of positive-strand RNA1 produced by T7 polymerase was only detected in long exposures. An additional band, not reported in infected cells, was detected by the minus strand probe. Its origin is unknown, but replication systems can yield artefactual RNAs arising by various mechanisms [31,41]. 

### 3.2. Flock House Virus Replicates in Close Association with Outer Mitochondrial Membranes in BSR T7/5 Cells

**Figure 2:**
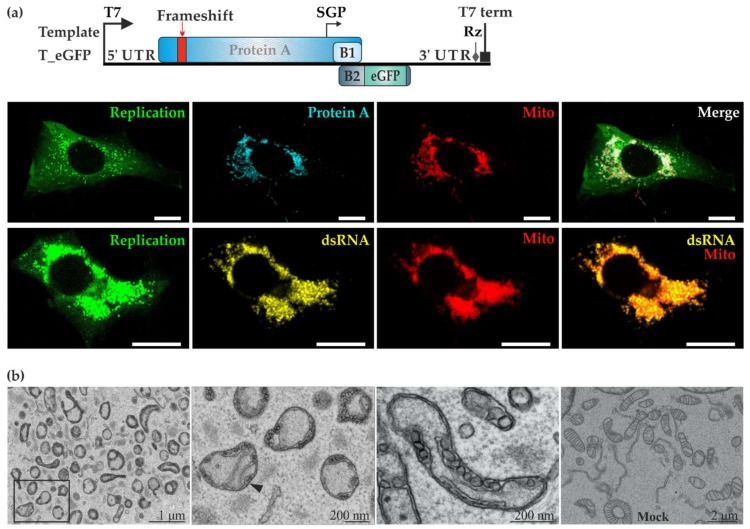
FHV uses the mitochondria of BSR T7/5 cells as replication niche. (**a**) The template T_eGFP expresses enhanced green fluorescent protein (eGFP) under the subgenomic promoter. BSR T7/5 cells were co-transfected with P_HA and T_eGFP. In the upper row, protein A (shown in cyan) was detected with anti-HA antibodies whereas mitochondria (shown in red) were detected with anti-Tom20 antibodies. In the lower row, double-stranded RNA (dsRNA, shown in yellow) was detected together with Tom20 (shown in red). In both rows, the cytoplasmic green fluorescence due to eGFP is shown on the left and the merged image on the right. The scale bars are 10 µM. (**b**) BSR T7/5 cells showing active replication were identified by green fluorescence and analyzed using correlative light electron microscopy (CLEM). Spherules were observed on the outer mitochondrial membranes in RNA replicating cells, as shown in different magnifications (three panels on the left). The black arrowhead indicates the neck structure of a spherule. No spherules were detected in cells transfected expressing P_GAA_Vis (which produces a red fluorescent marker from a second open reading frame) and T_eGFP (rightmost panel).

To better visualize FHV replication using microscopic techniques, the template plasmid T-eGFP was created by substituting Rluc with eGFP, under the control of the subgenomic promoter (Figure 2a). Following co-transfection with P_HA and T_eGFP, BSR T/5 cells with active trans-replication displayed strong green fluorescence. Using antibodies, we observed a close association between protein A and mitochondria as shown in Figure 2a. Secondly, the co-localization of dsRNA and the mitochondrial marker Tom20 clearly showed that mitochondria are the site of RNA replication for FHV in BSR T7/5 cells. The same combination of replicase and template plasmid constructs were used in CLEM experiments where replication-positive cells were identified by eGFP fluorescence and the samples were then further processed for electron microscopy. Mitochondria had undergone severe morphological changes during FHV replication (Figure 2b). The cristae were barely visible, and plentiful spherule invaginations were observed arising from the outer membrane. In contrast, cells co-transfected with the polymerase defective replicase plasmid and template showed healthy looking mitochondria (Figure 2b bottom right panel).

### 3.3. In Vitro Replication Based on the Trans-Replication System Yields Positive-Strand RNA

**Figure 3:**
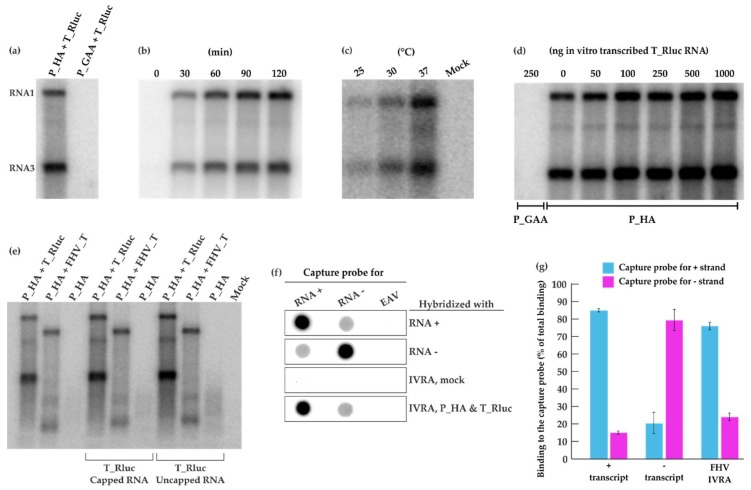
FHV RNA replication in vitro. (**a**) BSR T7/5 cells were transfected with P_HA + T_Rluc and P_GAA + T_Rluc. A crude mitochondrial pellet (CMP) was isolated by differential centrifugation 40 h post-transfection. CMP was incubated in an in vitro replication assay (IVRA) for 90 min at 30 °C and the RNA products were analyzed by electrophoresis and phosphorimaging. (**b**) The IVRA reactions from P_HA + T_Rluc transfected cells were incubated for different time periods. (**c**) The IVRA reactions were incubated at different temperatures for 90 min. (**d**) Different amounts of in vitro transcribed T_Rluc RNA were added to the IVRA performed as in panel (**a**). (**e**) CMP preparations from cells transfected with P_HA alone or with P_HA + T_Rluc (as before) or with P_HA + a shorter template FHV_T (lacking the marker gene) were analyzed for IVRA activity as in panel (**a**). The reactions were supplemented with 250 ng in vitro transcribed T_Rluc RNA (capped or uncapped), as indicated. (**f**) RNA probes recognizing positive or negative T_Rluc strands were transcribed in vitro and immobilized on membranes, followed by hybridization with IVRA from P_HA + T_Rluc transfected and mock cells. Equine arteritis virus (EAV) transcript was used as a control. (**g**) Binding of T_Rluc plus and minus transcripts as well as IVRA products to the capture probes was quantified in two independent experiments. Blue indicates binding to the capture probe for the plus strand and magenta to the capture probe for the minus strand.

Following co-transfection of P_HA + T_Rluc in BSR T7/5 cells, a crude mitochondrial pellet fraction was isolated by differential centrifugation and analyzed in an in vitro replication assay (IVRA). We observed a re-activation of viral RNA replication with high synthesis of both genomic RNA1 and subgenomic RNA3 (Figure 3a) when the reaction was allowed to proceed for 90 min at 30 °C. Clearly, the replication complexes formed in cells remained active following crude mitochondrial pellet (CMP) isolation and were able to replicate the T_Rluc template provided during transfection. As expected, the polymerase-defective replicase control did not display any activity. RNA products were already generated within the first 30 min of the reaction (Figure 3b). The reaction conditions used in the IVRA were based on previous studies done using FHV [36] or alphaviruses [42,43]. Additionally, we wanted to investigate the effect of varying temperatures on FHV replication in vitro. At 25 °C, visibly fewer RNA products were generated whereas a considerable boost in RNA synthesis was observed at 37 °C compared to IVRA at 30 °C (Figure 3c). In these experiments, the endogenous template present in the CMP was replicated. Adding additional in vitro transcribed template had a slight boosting effect on the activity, especially when added at high concentrations (Figure 3d).

To verify, whether the exogenously added template was able to replicate, we prepared a template of different length. In the template FHV_T, the reporter gene has been deleted, and thus it is somewhat shorter than T_Rluc, and corresponds to RNA1 (Figure 1a). FHV_T was also able to replicate under the IVRA conditions, when it had been originally transfected to the cells (Figure 3e, second lane). As expected, a CMP preparation containing protein A only did not show activity in the IVRA. We then added in vitro transcribed T_Rluc RNA (either capped or not capped during the transcription reaction) into these three types of CMP preparations. However, no additional bands were detected (Figure 3e), and thus we conclude that the CMP preparations can only replicate the endogenous template. This result has been reproduced with several protein A preparations not containing any endogenous template. 

To study the polarity of the FHV RNA synthesized, ^32^P-labeled IVRA products were hybridized with a membrane containing capture probes specific to FHV RNA of positive or negative polarity and a capture probe corresponding to EAV genomic fragment as a negative binding control. (Figure 3f). The IVRA products from mock samples did not show significant binding while the RNA synthesized in vitro by the isolated CMP strongly hybridized with the capture probe specific for the plus-strand RNA. Minor binding to the capture probe specific for the minus-strand RNA was observed (Figure 3f). ^32^P-labeled plus- and minus-RNA transcripts of the FHV template showed binding to both capture probes and thus the signals were quantified (Figure 3g). A strong hybridization was detected with the probe recognizing the plus strand and RNA synthesized in the IVRA from P_HA + T_Rluc (76%). The signal observed by the probe recognizing the minus strand (24%) was similar to the binding of minus transcript and therefore considered as background binding. This demonstrates that RNA products produced in vitro are mainly of positive polarity.

### 3.4. Mutations in the RNA Capping Enzyme Domain of Flock House Virus Protein A Strongly Affect Replication

**Figure 4:**
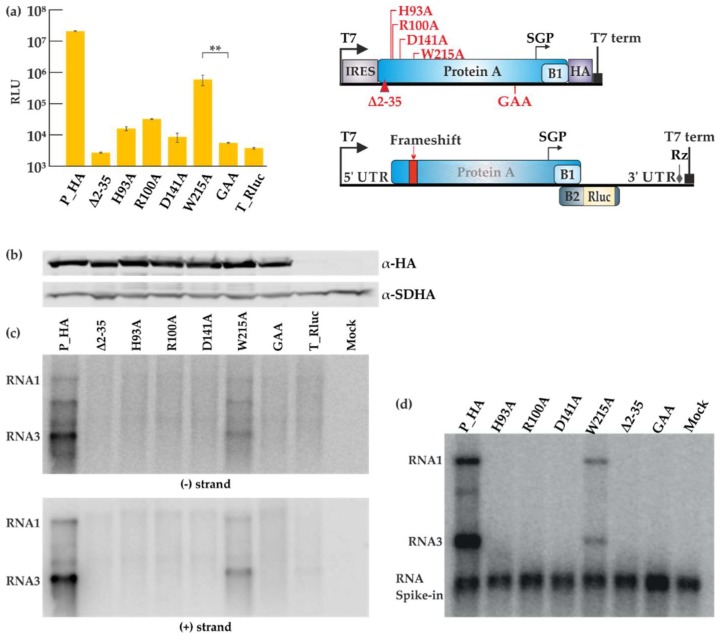
Mutational analysis of the FHV RNA capping enzyme. (**a**) BSR T7/5 cells were co-transfected with each FHV protein A mutant construct and template construct T_Rluc. Rluc activity was measured 40 h post-transfection. Mock values have been subtracted, error bars represent the standard deviation and ** designates *p* < 0.01 (Student’s *t* test). The location of the mutations is shown schematically on the right. RLU: relative light units. (**b**) Expression of protein A variants as detected by Western blotting with anti-HA antibodies; SDHA was used as a control. (**c**) Viral RNA synthesis detected by Northern blotting for minus and plus strands as indicated. (**d**) CMP fractions were isolated from transfected cells and used in the IVRA. The spike-in of short radioactive RNA was used to ensure equal isolation and loading of RNA. The relative density of the bands was measured with ImageJ software.

The role and importance of the putative RNA capping enzyme domain of protein A for FHV replication has not been studied in detail. We therefore individually mutated four of the most conserved amino acid residues in this domain, which were selected based on conservation in different nodaviruses, as well as between nodaviruses and the alphavirus superfamily [23]. The homology between the alphavirus and nodavirus enzymes is extremely low and based on family-level alignments, only these four residues can be considered to be properly conserved between the two groups. The putative roles of the residues are explained in the Discussion. The polymerase inactivating mutation (P_GAA), and a small deletion at the membrane binding site of protein A (Δ2-35) [44] were used as controls. The deletion mutant produced a very low amount of RNA in the yeast system [44], and the polymerase mutant is expected to have no replication activity. The sites of mutations are shown schematically in Figure 4a. Cells were co-transfected with the various replicase protein mutants and T_Rluc template, and the luciferase activity was measured (Figure 4a). All the capping mutants abolished viral replication except for the W215A mutant, which showed a reduction in luciferase activity. The mutations did not seem to affect the protein expression level as shown in Figure 4b. However, viral RNA synthesis was severely impaired, as detected for both negative and positive strand levels (Figure 4c), and only the W215A mutant showed significant RNA synthesis above the background. Finally, CMP fractions prepared from cells containing the mutant proteins and T_Ruc template were analyzed in IVRA. In concordance with the other results, only the W215A mutant was capable of in vitro RNA synthesis at reduced levels (Figure 4d). The mutant reproducibly synthesized a slightly lower proportion of RNA3 relative to RNA1, when compared to the wild type control reaction. 

**Figure 5:**
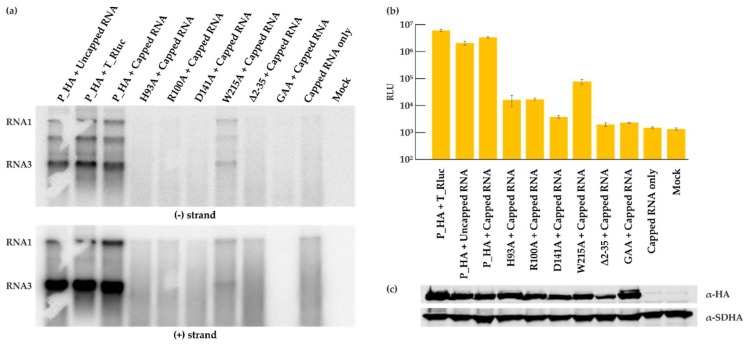
Capped RNA does not rescue the replication of the capping enzyme mutants. BSR T7/5 cells were co-transfected with the indicated FHV protein A constructs and either T_Rluc DNA or an in vitro transcribed FHV_T RNA template (capped or uncapped as indicated). (**a**) Viral RNA synthesis was detected by Northern blotting for minus and plus strands. (**b**) Rluc activity was measured 40 h post-transfection and the error bars represent the standard deviation. The data shown is a representative experiment. (**c**) Expression of protein A as detected by Western blotting with anti-HA antibodies; SDHA was used as a control. Note the slightly different sample order between panel (**a**) and panels (**b**) and (**c**).

To understand the action of the mutants further, we studied whether their replication activity could be rescued by the transfection of capped RNA template for replication. For wild type protein A, replication was equally efficient, when the template was provided from DNA (as before) or by transfection of either capped or uncapped RNA (Figure 5a, the first three lanes). When the mutant proteins were provided with capped RNA, they did not display any additional replication activity, and only the mutant W215A showed a low level of RNA synthesis (Figure 5a). Luciferase activity results were in concordance with the RNA synthesis (Figure 5b), and Western blotting confirmed the equal expression of the proteins, when capped RNA template was co-transfected with the protein expression plasmids (Figure 5c).

### 3.5. Comparison of Semliki Forest Virus and Flock House Virus Replication Systems

**Figure 6:**
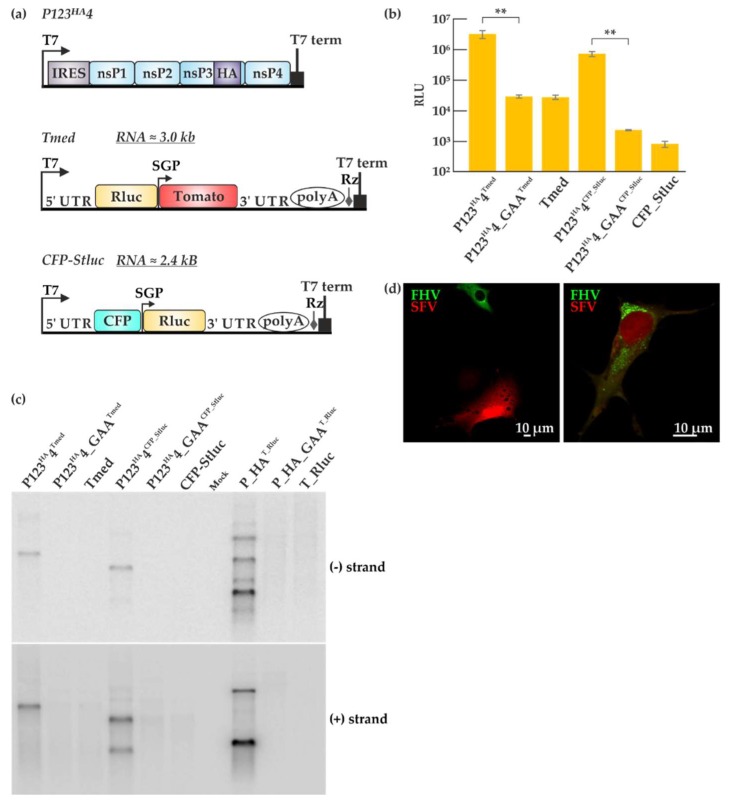
Comparison of Semliki Forest virus (SFV) and FHV trans-replication systems. (**a**) Graphic representation of the SFV trans-replication system. In the two templates used here, Rluc is placed under the genomic or subgenomic promoter. The abbreviations and symbols are as in Figure 1. Non-structural proteins (nsPs) 1–4 represent the SFV replicase proteins. Tomato and cyan fluorescent proteins (CFP) are the fluorescent markers present on the templates. (**b**) BSR T7/5 cells were transfected with the SFV trans-replication systems and incubated at 37 °C for 16 h. Luciferase signals are shown as means from two independent experiments and the mock values have been subtracted. Error bars represent the standard deviation. ** designates *p* < 0.01 (Student’s *t* test). The templates used are indicated as superscripts together with the replicase plasmids. (**c**) Viral RNA synthesis was detected by Northern blotting using probes designed to detect negative and positive strands, using probes for the Rluc encoding region, common to all the systems. (**d**) BSR T7/5 cells were transfected with the two replication systems together (P123^HA^4+ Tmed + P_HA + T_eGFP) using Lipofectamine at 28 °C and RNA replication was detected by microscopy 40 h post-transfection. Green fluorescence represents FHV replication while red fluorescence represents SFV replication.

Trans-replication systems have been used to study the alphaviruses SFV and chikungunya virus, of which SFV system is better characterized [30,31,39,45,46]. Therefore, we set out to compare the efficiency of SFV and FHV trans-replication systems. The plasmid constructs used for the SFV trans-replication system are depicted in Figure 6a. Non-structural proteins (nsPs) 1–4 constitute the viral replicase proteins required for SFV replication and are expressed from P123^HA^4, driven by the T7 promoter with nsP3 fused to the HA tag. Similar to the FHV virus polymerase-defective plasmid P_GAA, a SFV replicase construct P123^HA^4_GAA was created by mutating the catalytic domain of the SFV polymerase in nsP4. The Tmed template construct consists of Rluc under the T7 promoter and a Tomato fluorescent marker under the subgenomic promoter. In contrast, the CFP_Stluc template construct contains a cyan fluorescent protein (CFP) under the T7 promoter and Rluc under the subgenomic promoter. P123^HA^4 or P123^HA^4_GAA was co-transfected with Tmed or CFP_Stluc in BSR T7/5 cells and incubated for 16 h. Luciferase activities were high when P123^HA^4 and the templates were co-transfected whereas the P123^HA^4_GAA control only gave rise to background luciferase activity as shown in Figure 6b. As expected, a larger background was observed with Tmed as compared to CFP_Stluc because of the T7-driven transcripts generated in the cells can express luciferase in the former template. However, the fold amplification was rather similar. The FHV trans-replication (Figure 1c) showed a higher fold amplification than SFV (Figure 6b), but these values cannot be properly compared, as they are indirect measures requiring protein expression. Therefore, viral RNA synthesis of both trans-replication systems was analyzed in juxtaposition by Northern blotting (Figure 6c). After the transfection with either the SFV or the FHV trans-replication systems, the cells were lysed, RNA isolation was performed by phenol/chloroform extraction and an equal amount of total RNA (2 micrograms) was separated in a denaturing gel. Since we used probes recognizing a portion of the Rluc sequence, both genomic and subgenomic RNAs were detected for SFV co-transfections involving CFP_Stluc and FHV co-transfections and only the genomic RNA was detected with Tmed. Interestingly, the FHV trans-replication system was seen to generate larger amounts of viral RNA transcripts (Figure 6c). SFV appears to produce more genomic RNA, whereas FHV produces more subgenomic RNA.

Both SFV and FHV use cellular membranes for their replication [47]. To test whether SFV and FHV can replicate in the same cell, we transfected BSR T7/5 cells with P123^HA^4 + Tmed + P_HA + T_eGFP and looked for active viral replication by immunofluorescence 40 h post-transfection. The transfection efficiency was relatively low and some cells were seen to display either FHV or SFV replication as seen in Figure 6d (left panel). However, a few cells exhibited both green fluorescence, coming from the FHV trans-replication system, and red fluorescence, coming from the SFV trans-replication system (Figure 6d, right panel). This demonstrates that SFV and FHV trans-replication systems can function in the same cell to some extent, inducing reporter gene expression from their respective templates. Interestingly, there appeared to be a reduction in green and red fluorescence when both systems are active in the cell, which can translate to a reduction in replication activity compared to having only one trans-replication system active at a time. The low transfection efficiency limits the co-replication experiments, but it should be noted that for comparative studies of FHV and SFV replication, it is sufficient that the replication is observed in parallel experiments using each system individually. 

## 4. Discussion

Previous work has established that FHV RNA replication can take place in baby hamster kidney cells and their derivatives, the T7 polymerase-expressing BSR T7/5 cells [13,48]. Since FHV RNA2 is always replicated in trans by protein A, it could be assumed that the expression of protein A can also be uncoupled from the replication of RNA1. In fact, such a trans-replication system for RNA1 replication has been used to analyze the host factors required for FHV replication in yeast cells [14]. In contrast, the trans-replication system described here utilizes plasmid vectors in BSR cells (Figure 1), thus creating a system equivalent to the powerful alphavirus replication system we have reported previously [30].

This system yields high levels of FHV RNA synthesis and amplification, exceeding the level of alphavirus RNA, and prominent spherules on mitochondrial surfaces (Figure 2 and Figure 6). The system can be used as a basis for in vitro replication experiments, in which, similar to alphaviruses, mainly positive strand RNAs are produced (Figure 3) [34,49]. In contrast to a recent study [36], we find that the endogenous template present inside the cells is sufficient for replication activity in vitro. As a side note, we have used PEI as a transfection reagent for FHV and it was seen to give good transfection results. During transfection, DNA and PEI form polyplexes, bind to syndecans, are internalized by the clathrin-dependent or caveolae-dependent pathway, and are transported to late endosomes. The cell line and the nature of the polyplex determine which pathway is used most efficiently [50]. In contrast, the alphavirus system in BSR cells favors lipofectamine to PEI. This disparity could be due to the different replication sites (plasma membrane for alphaviruses and mitochondria for FHV) [19,51] or other factors related to the replication of the two viruses.

We have kept the BSR cells at 28 °C following transfection with the FHV replication system, as previously reported [48], because we have observed that incubation at 37 °C after transfection did not yield replication. It seems contradictory that no RNA replication was detected when cells were kept at 37 °C, but replication in vitro was very efficient at this high temperature (Figure 3c). This could suggest that the assembly of the replicase is temperature-sensitive, but when assembled, its function can be maintained at higher temperatures. However, a recent study suggests the intriguing alternative explanation that mitochondria usually operate at considerably higher temperatures compared to the cell cytoplasm [52]. Thus, the contradiction could be resolved, if the mitochondria (the replication sites) inside cells incubated at 28 °C, in fact, are operating at significantly higher local temperatures.

Although the nodavirus RNAs are capped, and the N-terminal domain of protein A is related to known capping enzymes, the capping enzyme activity of protein A has not been reported [23,53,54]. So far, our own attempts to demonstrate either the RNA capping or related guanine-7-methyltransferase activities for protein A have not been successful. Therefore, to start analyzing the significance of this enzyme for RNA replication, we mutated the most conserved amino acids of the capping domain. The sequence conservation between the nodavirus and alpha-like virus capping enzyme domain is quite limited [23], and so only four sites were initially selected for mutation. H93 corresponds to the proposed covalent guanylate binding site during RNA capping [55,56] and R100 is a highly conserved charged residues whose function remains uncertain [23]. The D141 residue is proposed to bind the methyl donor *S*-adenosylmethionine [55], and W215 corresponds to a conserved essential tyrosine in alphaviruses [23,55,57]. Of these, the W215A mutant is active in RNA synthesis at reduced levels (Figure 4), and so it seems that this residue is less important for FHV than the conserved tyrosine is in alphaviruses [33,57]. Interestingly, the three other FHV mutants fail to make any RNA (Figure 4), whereas the corresponding alphavirus and brome mosaic virus (BMV) mutants are active in at least minus strand synthesis [33,58]. This indicates mechanistic differences in RNA synthesis/RNA capping in the two virus groups. Furthermore, transfection of capped RNA template cannot rescue the replication activity of the mutants (Figure 5). It is currently unknown, why mutations at the putative capping enzyme active site seem to prevent all forms of FHV RNA synthesis. It is possible that the capping enzyme would be involved in the RNA binding/recruitment of the positive-sense RNA when the replication complexes are formed. There are other possibilities, such as the tight coupling of the RNA capping step and RNA replication, or very high sensitivity of the uncapped RNAs for degradation, but as mechanistic knowledge of the FHV polymerase remains sparse, these and further possibilities remain speculative. This result is a good example of the value of trans-replication systems in functional analysis, since the capping enzyme mutants are simply lethal in alphavirus genomes, precluding further experiments [57]. The RNA capping enzyme of the alpha-like group is a promising antiviral target due to the enzyme’s unusual mechanism [24,59], and, therefore, it is important to continue the structural and functional analysis of this viral enzyme family.

Beyond the individual enzymes, the entire replication complex or spherule is of general interest from the perspective of virus evolution, as explained in the Introduction. SFV and FHV use different cellular compartments for their replication [19,51]. The low-resolution structure of FHV replicase was recently revealed to be a ‘crown’ of twelve-fold symmetry located at the neck of the spherule [60]. It would be important to analyze whether the alphavirus-like spherules have any similarity to this structure, and whether similar host factors are required for spherule biogenesis in these two groups of viruses. Based on luciferase activity and detection of viral synthesis by Northern blotting, we established that the FHV trans-replication system is more efficient than the SFV trans-replication system (Figure 6b,c). We wondered whether both systems could coexist in the same cell. Hence, both the SFV and FHV trans-replication systems were co-transfected using lipofectamine and incubated at 28 °C. While this temperature is optimal for FHV replication, it slows down but does not block SFV replication [61]. Three clear phenotypes were observed (Figure 6d): FHV only replicated, SFV only replicated, or both viruses replicated in the same cell, although only a few cells exhibiting both green and red fluorescence were identified, which could be due to the low transfection efficiency. When we attempted CLEM with the same set of constructs, we could not observe FHV spherules (in mitochondria) together with SFV spherules (on the plasma membrane) in the same cell. A possible explanation would be that usually only one system could establish a high-level replication in an individual cell.
